# Epidemiology of invasive meningococcal disease in the United States: review of recent data and identified risk factors

**DOI:** 10.3389/fpubh.2026.1694023

**Published:** 2026-03-04

**Authors:** Jessica Presa, Daniel Spitz, Paul Balmer, Vincenza Snow, Kathleen Dooling

**Affiliations:** US Medical Affairs, Vaccines and Antivirals, Pfizer Inc, Collegeville, PA, United States

**Keywords:** epidemiology, high-risk group, invasive meningococcal disease, surveillance, vaccines

## Abstract

**Introduction:**

Tracking the spread of invasive meningococcal disease (IMD) in the United States is important for identifying risk factors and devising public health strategies to prevent infection.

**Methods:**

The epidemiology of IMD in the United States before, during, and after the COVID-19 pandemic (2016–2024) was assessed using surveillance data from the National Notifiable Diseases Surveillance System (NNDSS) and the Enhanced Meningococcal Disease Surveillance program (EMDS).

**Results:**

IMD case numbers declined during the pandemic (2020–2021) to 208 in 2021 but rebounded to 312 in 2022 and have continued to increase through 2024 (provisionally 477 cases). In 2022, serogroup C was the predominant serogroup (107 cases), followed by serogroup B (61 cases). Except during the pandemic, IMD cases were higher among those attending versus not attending college. During and after the pandemic, groups with the highest IMD incidence were those <1 year of age (range, 0.38–0.56 cases per 100,000 persons) and within the Black population (range, 0.09–0.19 cases per 100,000 persons). The percentage of IMD cases occurring after the pandemic in men who have sex with men and those with HIV increased substantially from during the pandemic. The percentage of IMD cases that occurred among people experiencing homelessness (PEH) was relatively high, ranging from 2.4–6.3%.

**Conclusion:**

The data indicate a rebound in IMD after the COVID-19 pandemic, highlighting the importance of strengthening surveillance and vaccination among high-risk populations.

## 1 Introduction

IMD is a relatively rare but severe infectious disease, characterized by rapid clinical progression and often severe or fatal outcomes (1, 2). The Centers for Disease Control and Prevention (CDC) has reported a case-fatality rate for IMD of 10–15%, even with antibiotic treatment (3), and ≤20% of hospitalized patients with IMD have lifelong disabling sequelae (4). IMD incidence peaks in infants and young children, with secondary peaks in adolescents/young adults and sometimes older adults (4). Among 12 identified meningococcal serogroups, five are vaccine-preventable in the United States (A, B, C, W, and Y), with serogroups B, C, W, and Y accounting for most IMD cases; serogroup A is not currently endemic in the United States (3, 4). IMD is largely preventable through vaccination (5). The US Advisory Committee on Immunization Practices (ACIP) recommends vaccinating with meningococcal serogroups A, C, W, Y conjugate vaccine (MenACWY) at age 11 or 12 years and again at 16 years (6). Additionally, people from the ages of 16–23 years are recommended to receive a meningococcal serogroup B vaccine (MenB) based on shared clinical decision-making (6). The ACIP also recommended MenACWY and MenB for persons aged ≥2 months and ≥10 years, respectively, at increased risk for meningococcal disease (6).

The epidemiology of IMD constantly evolves and varies over time and across different regions (4). Despite the introduction of new meningococcal vaccines over the last few decades, and the periodic updating of vaccination recommendations, IMD outbreaks persist in at-risk populations in the United States (7, 8). In the United States, overall incidence of IMD has been declining since the year 2000, reaching historic lows during the COVID-19 pandemic (9, 10). However, sharp post-pandemic rebounds in IMD, particularly serogroup Y disease, have been observed (9). Demographic groups with a higher incidence of IMD in the United States are those who are between the ages of 30–60 years, Black, and adults with HIV (9). Elevated IMD incidence has also been reported in persons with complement component deficiency and functional or anatomic asplenia and in men having sex with men (MSM). The ACIP has recommendations for MenACWY and MenB vaccination for people in these groups with high incidence of IMD (6).

Tracking the epidemiology of IMD can aid in the identification of outbreaks and help inform public health strategies for preventing disease (4). In the United States, IMD is a reportable condition tracked by the CDC using the National Notifiable Diseases Surveillance System (NNDSS) (11). To improve surveillance of IMD, a comprehensive Enhanced Meningococcal Disease Surveillance (EMDS) program was implemented in 2015 (9, 12) that collects more complete data than the NNDSS on key variables for monitoring meningococcal disease epidemiology. Also, a component of the CDC’s Emerging Infections Programs, Active Bacterial Core surveillance, an active laboratory- and population-based surveillance system, conducts surveillance in 10 states for invasive bacterial pathogens, including IMD (13). Across these reporting systems, IMD outbreaks are reported and pertinent information on those with IMD is gathered, including age and racial group and the percentage that have high-risk conditions, attend college, are people that are experiencing homelessness (PEH), have HIV, or are MSM (9).

This narrative review summarizes recent data regarding IMD gathered from multiple surveillance systems to better understand the current US epidemiology of IMD.

## 2 Data sources and approach

This is a narrative review of the epidemiology of meningococcal disease in the United States. In the United States, meningococcal disease is reportable to local and state health departments and tracked by the CDC (9). The epidemiology of IMD (cases and/or incidence rates) was reviewed from 2016–2024 with data derived from the CDC’s NNDSS and EMDS (9, 11). The NNDSS collects and processes data for national notifiable diseases from approximately 3000 public health departments around the United States, which helps identify disease outbreaks and monitor shifts in disease patterns. EMDS collects isolates from all IMD cases and additional key demographic information for monitoring IMD epidemiology from health departments in all 50 states and 3 large jurisdiction urban health departments. In addition to the inclusion of CDC data sets, an extensive literature search of PubMed and review of CDC websites was performed to confirm that all relevant, available data were captured. Data extracted for this review included the epidemiology of all-type IMD and serogroup-specific IMD.

## 3 Results

### 3.1 IMD cases

**Figure 1:**
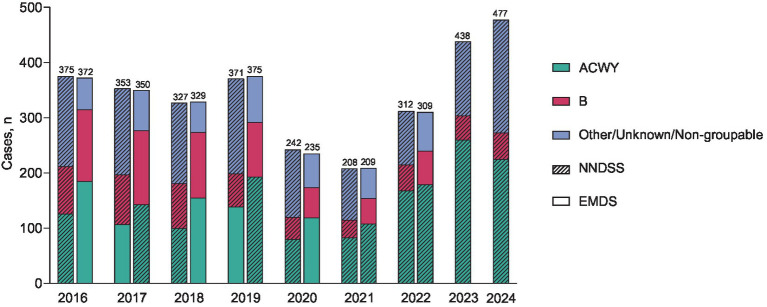
Number of invasive meningococcal disease (IMD) cases in the United States by serogroup annually from 2016–2024 reported by the Nationally Notifiable Diseases Surveillance System (NNDSS) and Enhanced Meningococcal Disease Surveillance (EMDS) (14). EMDS data for 2023 and 2024 are not yet available. Case counts reported by the NNDSS for 2023 and 2024 are provisional and subject to change. ACWY = serogroups A, C, W, and Y; B = serogroup B.

**Figure 2:**
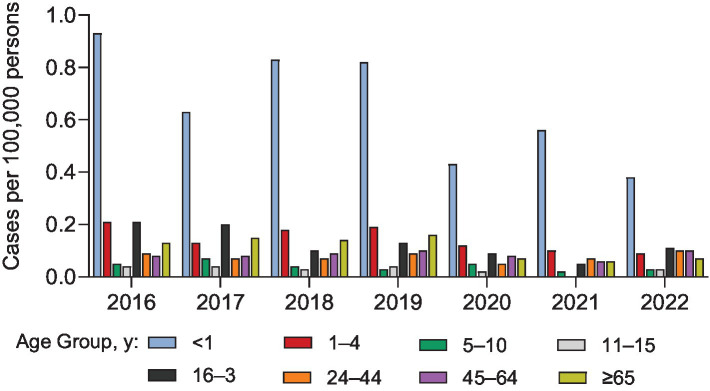
Annual incidence of invasive meningococcal disease (IMD) cases in the United States by age group from 2016–2022 reported by the Enhanced Meningococcal Disease Surveillance (EMDS) (14).

The NNDSS and EMDS reported similar IMD case numbers during 2016–2022 (Figure 1) (14). Cases were trending downward from 2016–2018 but in 2019 rebounded to numbers similar to those in 2016 (Figure 1). Substantial decreases in cases occurred during the COVID-19 pandemic (2020 and 2021) and then increased in 2022 and 2023 (NNDSS, 312 and 438 cases, respectively), exceeding pre-pandemic levels. NNDSS data indicate that there were 67 (18.1%) more cases in 2023 than in the pre-pandemic year 2019, with this rebound further increasing in 2024 (provisionally 477 cases; Figure 1). Yearly incidence of IMD from 2016 through 2022 was greatest in infants <1 year of age and lowest in children 5–10 and 11–15 years of age (Figure 2) (9, 14).

Isolates for 2023 and 2024 are still being processed and more accurate serogrouping data will be released with the respective EMDS reports, however, as reported by NNDSS, the most common serogroups that caused IMD in 2023 (59.4%) and 2024 (47.1%) were A, C, W, and Y combined (serogroup A is rarely reported in the United States), with a smaller percentage of serogroup B cases (10.0 and 10.1%, respectively; Figure 1) (6, 14). In 2022 (most recent EMDS data), the EMDS reported 107 cases of serogroup C (34.6% of all cases reported), 12 cases of serogroup W (3.9%), 59 cases of serogroup Y (19.1%), 61 cases of serogroup B (19.7%), 40 other/unknown cases (12.9%), and 30 non-groupable cases (9.7%).

### 3.2 Risk factors for IMD

From 2016–2022, except for 2 years at the beginning of the COVID-19 pandemic (2020 and 2021), the incidence of IMD was substantially higher among 18- to 24-year-olds attending college versus those who were not (Table 1) (14). Even so, coverage rates for both MenACWY and MenB vaccination among those with IMD were higher for college students, except for MenB coverage in 2020, which was slightly higher among those not attending college (Table 1). Although outbreaks in college settings have become less frequent, they still occur, as seen in February-March 2022, with 3 serogroup B cases reported at Florida State University (15).

**Table 1:** IMD cases, incidence, and vaccination rates among 18- to 24-year-olds who are attending college versus not attending college reported by EMDS, 2016–2022^a^ (14).

Year	IMD cases, *n*	Incidence^b^	MenACWY vaccination rate among cases, %^c^	MenB vaccination rate among cases, %^c^
2016				
Attending college	25	0.23	92	7
Not attending college	9	0.05	44	0
2017				
Attending college	31	0.27	69	44
Not attending college	8	0.04	67	33
2018				
Attending college	11	0.10	94	14
Not attending college	9	0.05	75	0
2019				
Attending college	21	0.18	95	56
Not attending college	20	0.11	75	0
2020				
Attending college	10	0.09	86	14
Not attending college	17	0.09	69	18
2021				
Attending college	5	0.04	100	50
Not attending college	12	0.06	50	11
2022				
Attending college	24	0.20	76	36
Not attending college	18	0.09	60	13

Data from EMDS Reports (14). EMDS = Enhanced Meningococcal Disease Surveillance; IMD = Invasive meningococcal disease; MenACWY = Meningococcal serogroups A, C, W, Y conjugate vaccine; MenB = Meningococcal serogroup B vaccine.

a Assumes percentage of 18- to 24-year-olds attending college is 36.2% in 2016 and 38.3% in 2017–2022.

b Cases per 100,000 population.

c Among those with known vaccine history.

Incidence of IMD reported by NNDSS from 2016 through 2021 showed differences based on race (Supplementary Figure 1A) (14). From 2017 through 2022, rates were highest within the Black population (0.09–0.19 per 100,000 persons) and lowest among Asian or Pacific Islanders (0.01–0.06 per 100,000 persons; Supplementary Figure 1A). Incidences were generally similar between Hispanic and non-Hispanic groups, although in more recent years the trend has been toward higher rates in the Hispanic population (Supplementary Figure 1B). Since the COVID-19 pandemic, numerous cases of IMD caused by serogroup Y have occurred in racial and ethnic minorities. The CDC identified 29 cases caused by ciprofloxacin-resistant serogroup Y strains during 2019–2021, 24 of which were also penicillin resistant (16); 20 (69%) of these cases occurred in Hispanic and Latino groups. An outbreak of 39 cases caused by serogroup Y was reported in Virginia from June 2022–March 2024 that disproportionally affected Black individuals and those with HIV (17, 18). There have been other recent outbreaks, one caused by serogroup B (6 cases) within the Amish community in Ohio from December 2023-January 2024 and one caused by serogroup C (2 cases and 1 death) among those at a correctional facility in Oklahoma from March through May 2024 (19). Also, two recent outbreaks of IMD caused by serogroup W disease have been reported: one in Iowa (12 cases and 2 deaths) from November 2022–July 2023 and another ongoing outbreak among people who traveled to the Kingdom of Saudi Arabia (14 cases) that began in April 2024 (19).

The EMDS reports demographic and clinical characteristics of IMD in those cases for which information is available (14). EMDS data show a high risk for IMD among MSM, PEH, and those with HIV infection (Table 2). From 2017–2022 the percentage of PEH among IMD cases ranged from 2.4–6.3% (Table 2). Three recent outbreaks caused by serogroup C have occurred among PEH: one in 2021 (3 cases) and two in 2022 (2 cases and 3 cases, respectively) (19, 20). In an ongoing outbreak that began in January 2024, 6 cases of IMD caused by serogroup Y have been reported among PEH in Denver (21).

**Table 2:** Yearly percentages of all reported IMD cases occurring within specific demographic groups and antibiotic resistance among serogroup Y cases^a^ reported by EMDS, 2016–2022 (14).

Year	PEH	MSM^b^	HIV infection	Complement inhibitor use	Serogroup Y ciprofloxacin- and penicillin-resistant
2016	NA	39.8	4.3	NA	NA
2017	2.4	18.9	9.9	0	NA
2018	5.2	6.0	2.8	1.2	NA
2019	2.6	9.9	2.9	1.8	14
2020	6.2	2.6	4.9	2.5	19.5
2021	6.3	23.7	7.3	0.7	30.8
2022	4.7	39.3	16.8	3.0	10.2

Data are reported as %. Data from EMDS Reports (14).

EMDS = Enhanced Meningococcal Disease Surveillance; IMD = Invasive meningococcal disease; MSM = Men who have sex with men; NA = Not available; PEH = People experiencing homelessness.

a IMD cases for which information is available.

b Men aged ≥16 years.

MSM and those with HIV are also at increased risk for IMD (6, 12). The incidence of IMD from 2015–2016 per 100,000 persons was previously reported to be 0.54 in MSM and 0.10 in non-MSM (12). Evaluation of IMD cases identified through Active Bacterial Core surveillance during 2009–2019 showed that the average annual incidence of IMD per 100,000 persons among those with HIV was 0.96 versus 0.16 among those without HIV (22). EMDS reported that among cases of IMD between 2016–2022, the percentage of those who were MSM ranged from 2.6–39.8% and the percentage of those with HIV ranged from 2.8–16.8% (Table 2). The percentage of MSM and those with HIV affected by IMD has gone up substantially from pre-COVID-19 pandemic rates of 9.9 and 2.9%, respectively, in 2019 to 39.9 and 16.8% in 2022. A recent outbreak of serogroup C IMD (44 cases, 9 deaths) occurred from November 2021–November 2022 in Florida predominantly among MSM, 34% of whom were HIV positive. In 2022, approximately half the cases of IMD in persons with HIV were related to an outbreak among MSM (Supplementary Figure 2) (23).

EMDS stated that 0–3.0% of people with IMD reported use of complement inhibitors in 2017–2022 (Table 2), suggesting that people taking complement inhibitors are at a much higher risk for IMD (24). Among IMD cases caused by serogroup Y from 2019–2022, 14.0–30.8% were resistant to ciprofloxacin and penicillin.

In 2021, coverage rates for adolescents receiving a first dose of MenACWY vaccination were 95.6, 89.2, and 90.2% of the Asian, White, and Black populations, respectively, and 86.5% of the Hispanic population (Supplementary Table 1) (25). Coverage for ≥1 dose of MenACWY was significantly lower among adolescents with no insurance (77.5%) compared with those who had only private insurance (90.2%) or any Medicaid insurance (88.8%; Supplementary Table 1) (25).

## 4 Discussion

The epidemiology of IMD is continually changing, and surveillance is important to inform public health policy and vaccination programs (4). During the COVID-19 pandemic, IMD incidence decreased globally and then began to rebound post-pandemic (26, 27). Review of data from NNDSS and EMDS show that these trends also occurred in the United States. Cases markedly decreased during the COVID-19 pandemic (2020-2021) from pre-pandemic levels (2016–2019) and then rebounded in 2022. In 2023, case numbers exceeded those from pre-pandemic periods, and NNDSS data from 2024 indicate that cases continue to rise. The incidence of IMD continues to be highest in the most vulnerable age group: infants <1 year of age. Nevertheless, the potential for underreporting of IMD in the United States should also be noted. A recent retrospective analysis of medical records from 988 patients with signs or symptoms of meningitis and/or encephalitis that were admitted to five hospitals during 2014–2023 identified several gaps in specimen collection and laboratory testing (28). The authors concluded that the gaps could lead to under-diagnosis of IMD, resulting in underreporting of IMD by public health surveillance. Additionally, meningococcal pneumonia might not be reported if *Neisseria meningitidis* is not isolated in the blood (29).

Increases in case numbers in 2022–2024 were predominantly due to serogroups C, W, and Y and unknown or non-groupable serotypes, whereas serogroup B accounted for only a minority (<20%) of total cases. In 2024, the CDC issued a health advisory to alert healthcare providers of an increase in IMD, mostly attributable to serogroup Y (3). It was noted that the US case-fatality rate for the serogroup Y disease reported through 2023 was 18%, which is higher than the US case-fatality rate of 11% for serogroup Y in 2017–2021. Factors contributing to the continuing increase in IMD after the COVID-19 pandemic are uncertain. Known risk factors for IMD include age, chronic underlying illness, crowded living space, and active and passive smoking (30). A previous analysis of IMD incidence among persons aged 18–24 years in the United States in 2014–2016 found that those attending college were at significantly higher risk for serogroup B disease than those not attending college (30). Recent EMDS data indicate that in 2020 and 2021, during the COVID-19 pandemic, incidence of IMD was similar between those attending college and those not attending college; however, in 2022 the incidence was twice as high in those attending college, predominantly due to serogroup B, which was 0.10 versus 0.03 per 100,000 persons in those attending versus not attending college, respectively. This was despite a higher rate of meningococcal vaccination among those attending college.

A recent meta-analysis found that testing positive for HIV was associated with a substantially elevated risk for IMD (30). EMDS data showed that the percentage of IMD cases occurring in those with HIV rose from <10% in 2016–2021 to 16.8% in 2022. The percentage of IMD cases that occurred among MSM also rose substantially from a low of 2.6% in 2020 to 39.3% in 2022. The factors that contribute to the higher rates of IMD, and outbreaks, among MSM are uncertain, but have been suggested to be increased close social networks, increased number of social contacts, and non-specificity of flu-like symptoms that impede recognition of disease (31). Generally, IMD outbreaks occur in organizational settings such as dormitories, army barracks, and schools, or mass gatherings such as social gatherings or in villages (32). In a 2021-2022 outbreak of serogroup C IMD among MSM in Florida, 34% of those affected were HIV positive (33). In 2016, the ACIP recommended people with HIV be routinely vaccinated with a MenACWY vaccine (6). A recent nationwide study in the United States found that only 16.3% of those with a new diagnosis of HIV had received the MenACWY vaccine during the 24 months after their diagnosis, suggesting a need to educate patients and healthcare providers about the role of meningococcal vaccination in high-risk individuals (34). Results from a US survey of MSM published in 2019 found that common barriers to meningococcal vaccination among MSM were lack of insurance or ability to pay and distrust of the medical system (31).

PEH are at higher risk for IMD than those who are not, and the risk for outbreaks is higher within that population (35, 36). It has been suggested that crowded living shelters and a high prevalence of comorbid conditions contribute to the increased risk for infectious diseases among PEH (35). Rudmann et al. (35) reported that, from 2016–2019, the annual incidence of IMD in the United States was 2.12 versus 0.11 per 100,000 persons among PEH versus those not experiencing homelessness, respectively (i.e., **~**19 times higher in PEH). Also, an analysis of insurance claims data from 2016–2019 found that, among people with Medicaid, IMD incidence was 3.17 versus 0.12 cases per 100,000 person-years in PEH versus those not experiencing homelessness (36). Recent EMDS data support a higher risk for IMD among PEH; percentages of IMD cases that occurred in PEH ranged from 2.4–6.3% in 2017–2022.

More broadly, the risk of IMD is higher in low-income populations. Globally, communities with low socioeconomic status have been found to have the highest risk of IMD and the lowest uptake of meningococcal vaccines (37). In the United States, analysis of insurance claims data from 2016–2019 found that IMD incidence was higher among persons enrolled in Medicaid compared with persons with commercial insurance across all age groups except those 15–24 years of age. Incidence of IMD was also significantly higher among those receiving Medicaid whose family income was 0–100% of the federal poverty level versus those whose income was 101–400% of the federal poverty level (36).

In the United States, data show a significant difference in health and healthcare status among racial and ethnic minorities (38). Historically, the risk for IMD was higher in Black versus White populations; however, data from 2004–2007 reported similar incidence rates between these groups (39). While there are a small number of IMD cases and small differences in cases can have a large impact on incidence, recent data from 2017–2021 indicate that across racial groups in the United States, IMD incidence was consistently highest in the Black population and in 2021 (the most recent year with available data) was approximately twice as high as in the White population. A recent systematic analysis showed that meningococcal vaccination coverage in adolescents and young adults is affected by racial/ethnic, socioeconomic, and geographic disparities in the United States, with gaps existing in insurance coverage for vaccines in these populations (40).

Recent data from the CDC indicate that adolescent vaccination coverage with the MenACWY vaccine in 2021 was similar among the Black, White, and Hispanic populations, but higher in the Asian population (25). In a 2016 survey, 57% of parents/legal guardians of 16- to 19-year-olds (weighted percentage based on US population distribution) were unaware of MenB vaccines (41), with greater unawareness among Hispanic and non-White populations. Of those who were unaware of MenB vaccines, 36% reported interest in seeking MenB vaccine information, with the highest interest among those of Hispanic ethnicity (41). A 2019 IMD awareness survey of parents/guardians across numerous countries, including the United States, reported that parents generally had an understanding of IMD; however, parents were much less aware of the different IMD serogroups and the vaccines that are needed to fully protect against all serogroups (42).

Exposure to cigarette smoke is also a known risk factor for IMD (36, 43). Although tobacco use has declined in the United States over the last several decades, in 2021, approximately 18.7% (46 million) of US adults used some tobacco product (44). Cigarettes were the most frequently used tobacco product (11.5%), followed by e-cigarettes (4.5%) (44). From 2020-2021, the prevalence of cigarette smoking decreased while the prevalence of e-cigarette use increased (44). In 2023, among US middle school and high school students, 2.13 million (7.7%) used e-cigarettes, with 25.2% of those students reporting daily use (45). E-cigarettes, which share some parallels to cigarettes, have numerous detrimental effects on the respiratory mucosa, such as impairing phagocytosis, which could result in impaired bacterial clearance, and induction of inflammation and disruption of the respiratory epithelium (46–48). Such damage to the mucosal layer could result in a higher susceptibility to respiratory infections, although further investigation is needed to determine if e-cigarette use does increase susceptibility to IMD. Additionally, it is possible that sharing of e-cigarettes could increase the risk of meningococcal transmission through increased sharing of respiratory secretions between individuals.

We note that this was a narrative review and descriptive in design; no inferential statistical analyses were performed. Although this narrative review provides a relatively comprehensive evaluation of recent epidemiology of IMD, obtained primarily from CDC data sets, it was not intended to be an exhaustive review of the current epidemiology of meningococcal disease in the United States.

In conclusion, cases of IMD have been increasing in the United States since an all-time low during the COVID-19 pandemic and currently exceed pre-pandemic levels. In particular, IMD incidence has trended higher in Black versus White populations and in Hispanic versus non-Hispanic White populations in recent years. These increases highlight the importance of meningococcal vaccination programs to help prevent IMD and the need to better inform patients, particularly those in high-risk groups who may have limited access to preventative care, and healthcare providers about the risk of meningococcal disease and means of prevention. Currently available MenACWY and MenB vaccines together provide broad coverage across these serogroups (6). Additionally, a pentavalent vaccine, MenABCWY, which provides protection against the 5 predominant serogroups, was licensed in the United States in 2023 (49). The surveillance data reported here indicate rising IMD incidence post-pandemic, emphasizing targeted vaccination and monitoring in high-risk groups.

## Data Availability

Publicly available datasets were analyzed in this study. This data can be found here: Centers for Disease Control and Prevention. Meningococcal Disease Surveillance and Trends. Available from: https://www.cdc.gov/meningococcal/php/surveillance/index.html and Centers for Disease Control and Prevention. National Notifiable Diseases Surveillance System (NNDSS) 2025. Available from: https://www.cdc.gov/nndss/infectious-disease/index.html.
