# Supplementary material for: Epidemiology of invasive meningococcal disease in the United States: review of recent data and identified risk factors
Source: Front Public Health. 2026 Mar 4;14:1694023. doi: 10.3389/fpubh.2026.1694023 (PMC12996253; doi:10.3389/fpubh.2026.1694023)
Supplement: Supplementary file 1 [file Data_Sheet_1.pdf]

Supplementary Materials

Supplementary Table 1. Estimated MenACWY vaccination coverage among adolescents aged 13–17[a] years, by race and ethnicity and health insurance status in the United States, 2021 (1).

	Race and ethnicity, % (95% CI)						Health insurance status[d], % (95% CI)			
**Doses, n**	**American Indian / Alaska Native (n=211)**	**Asian (n=779)**	**Black (n=1,799)**	**White, non-Hispanic (n=11,256)**	**Hispanic (n=3,028)**	**Multiracial, non-Hispanic (n=1,214)**	**Private insurance only (n=11,378)**	**Any Medicaid (n=5,269)**	**Other insurance (n=1,312)**	**Uninsured (n=419)**
≥1	93.4 (87.3–96.6)	95.6 (93.5–97.0)[c]	90.2 (87.4–92.4)	89.2 (88.2–90.2)	86.5 (83.0–89.5)	88.1 (84.2–91.2)	90.2 (88.8–91.4)	88.8 (86.9–90.4)	85.5 (78.7–90.4)	77.5[c] (67.8–84.9)
≥2[b]	63.8 (36.9–84.1)	78.8 (65.5–88.0)[c]	61.7 (51.7–70.8)	61.3 (57.3–65.1)	50.6 (41.6–59.6)[c]	57.5 (43.6–70.4)	61.7 (57.3–65.9)	60.7 (54.7–66.4)	56.6 (45.1–67.5)	NA

CDC=Centers for Disease Control and Prevention; MenACWY=meningococcal conjugate vaccine; NA=not available.
Survey data are from the CDC TeenVaxView.
[a]Includes percentages receiving MenACWY and meningococcal-unknown type vaccine.
[b]≥2 doses of MenACWY or meningococcal-unknown type vaccine. Calculated only among adolescents who were aged 17 years at interview. Does not include adolescents who received 1 dose of MenACWY vaccine at age ≥16 years.
[c]Statistically significant difference (*P*<0.05) in estimated vaccination coverage by race/ethnicity or health insurance status; referent group was adolescents non-Hispanic white people or adolescents with private insurance.
[d]Adolescents' health insurance status was reported by parent or guardian. "Other insurance" includes the Children's Health Insurance Program, military insurance, Indian Health Service, and any other type of health insurance not mentioned elsewhere.

Supplementary Figure 1. Invasive meningococcal disease incidence by race (A) and ethnicity (B) from 2016–2022 reported by the Nationally Notifiable Diseases Surveillance System (2).

A.

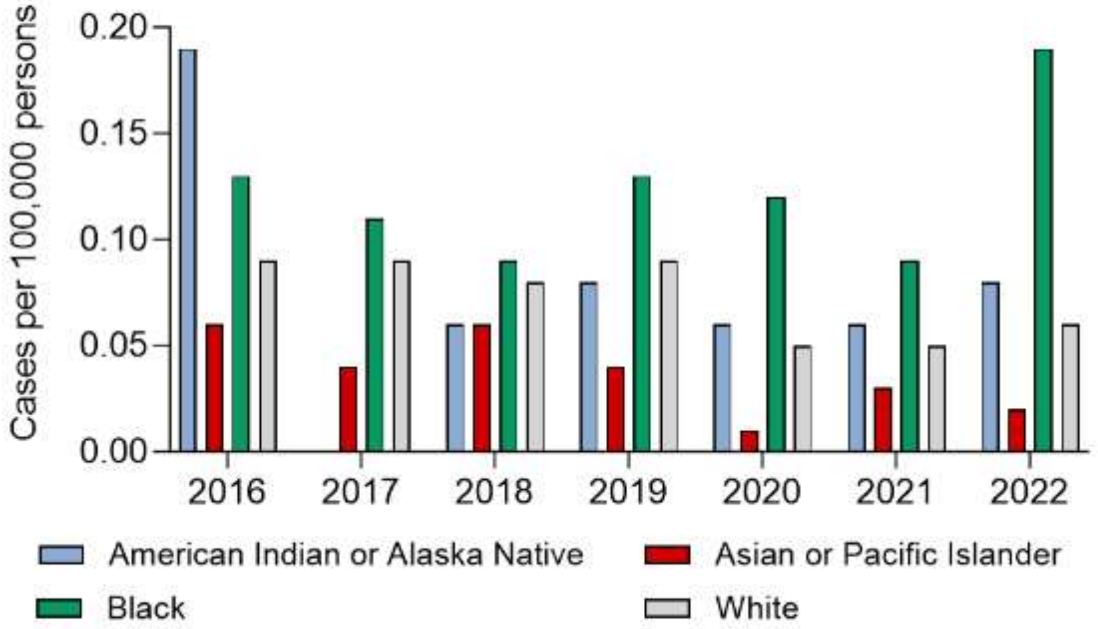

B.

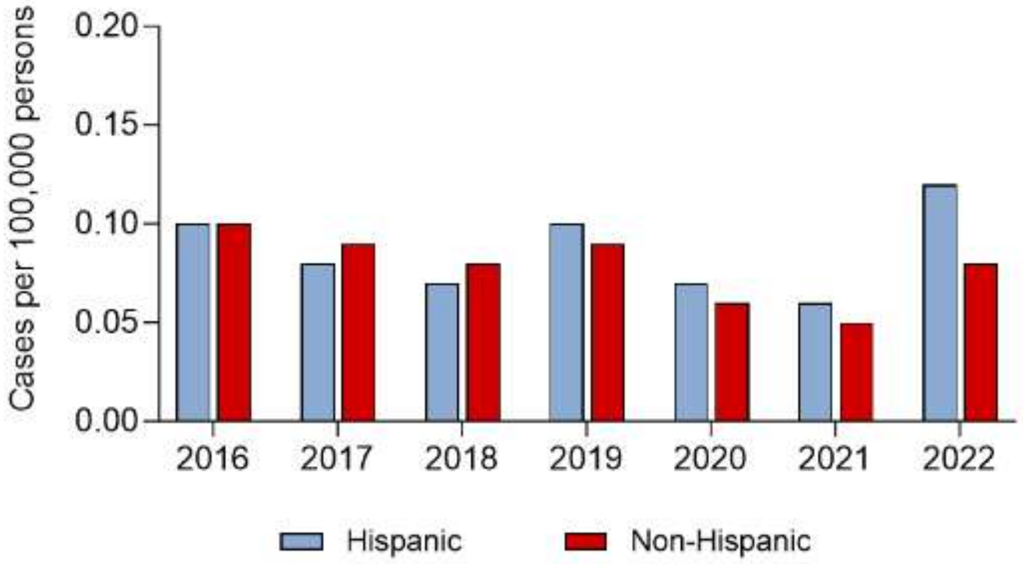

Supplementary Figure 2. Cases occurring during known outbreak of invasive meningococcal disease among persons with HIV in the United States from 2017–2022 (3). MSM=men having sex with men.

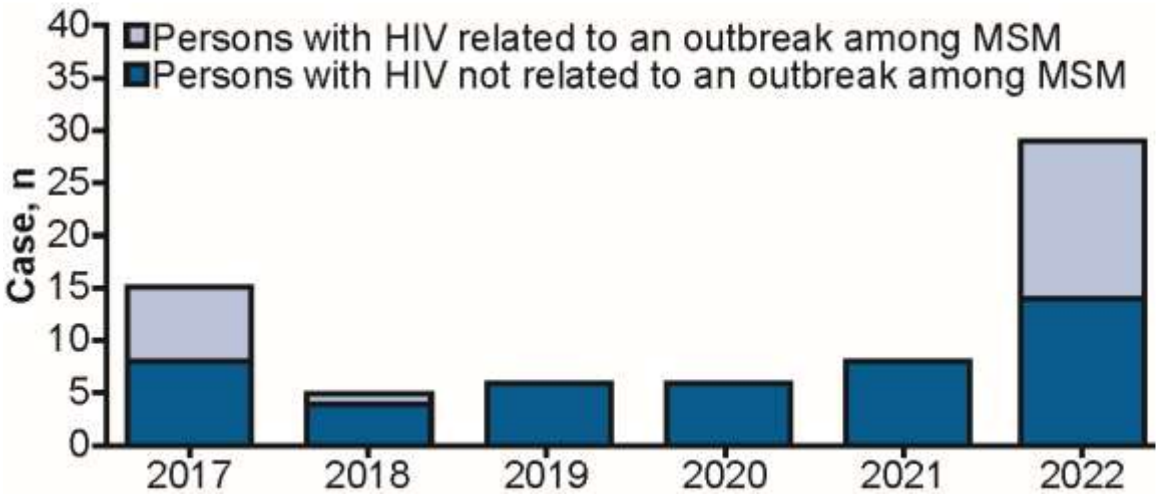

References

1. Centers for Disease Control and Prevention. TeenVaxView 2024. Available from: https://www.cdc.gov/teenvaxview/?CDC_AAref_Val=https://www.cdc.gov/vaccines/imz-managers/coverage/teenvaxview/index.html.

2. Centers for Disease Control and Prevention. CDC Stacks Public Health Publications 2024. Available from: https://stacks.cdc.gov/welcome.

3. Rubis AB, Howie RL, Marasini D, Sharma S, Marjuki H, McNamara LA. Notes from the field: increase in meningococcal disease among persons with HIV - United States, 2022. MMWR Morb Mortal Wkly Rep. 2023;72(24):663-4. doi:10.15585/mmwr.mm7224a4.
